# Advances in porphyrin-based photosensitizers for photodynamic therapy of A549 lung cancer

**DOI:** 10.3389/fphar.2026.1878210

**Published:** 2026-06-30

**Authors:** Guoxiang Rong, Fengxi Li, Ting Li, Huoying Pan, Xuanxuan Luo, Xiaohua Zheng

**Affiliations:** 1 Department of Cardiothoracic Surgery, Affiliated Danyang Hospital of Nantong University, Danyang, China; 2 Medical School of Nantong University, Nantong, China; 3 School of Pharmacy, Nantong University, Nantong, Jiangsu, China

**Keywords:** A549, combination therapy, porphyrin, reactive oxygen species (ROS), tumor microenvironment

## Abstract

Porphyrin-based photosensitizers are widely used in photodynamic therapy (PDT) for A549 lung cancer. However, their clinical efficacy is limited by poor water solubility, the trade-off between fluorescence emission and singlet oxygen generation, and insufficient tumor targeting. To address these issues, researchers have turned to molecular design strategies. By constructing donor–acceptor systems and employing supramolecular assembly, they have significantly improved the photophysical properties of these photosensitizers. Building on these advances, smart delivery systems responsive to multiple tumor-specific stimuli, such as overexpressed matrix metalloproteinases, hypoxia, and acidic pH in the A549 tumor microenvironment, have been developed. These systems enable precise targeting and on-demand activation of the photosensitizer. To overcome the limitations of monotherapy, recent studies combine PDT with chemotherapy, starvation therapy, or ferroptosis. Such combinations enhance cancer cell killing through cascade amplification or mutual reinforcement mechanisms. In parallel, innovative delivery platforms, including engineered probiotics, cell membrane-derived vesicles, and all-API (active pharmaceutical ingredient) nanoparticles, have greatly improved tumor accumulation and biocompatibility of porphyrin photosensitizers. This review systematically integrates these recent advances, providing a solid foundation for the clinical translation of porphyrin-based photosensitizers in precision PDT for A549 lung cancer.

## 1 Introduction

According to the World Health Organization, cancer is one of the leading causes of death worldwide (Siegel et al., 2024; Siegel et al., 2025; Wagle et al., 2025; Negahdary et al., 2026; Gao et al., 2026). Billions of dollars are spent each year on research aimed at overcoming this disease. Among all cancers, lung cancer is the most common and has consistently ranked highest in both incidence and mortality (Kratzer et al., 2024). Non-small cell lung cancer (NSCLC) accounts for approximately 85% of all lung cancer cases (Chen et al., 2022; Araghi et al., 2023; Su et al., 2025). The A549 cell line, a human-derived NSCLC adenocarcinoma model, is widely used to study lung cancer biology and test new therapies (Liu et al., 2025; Meyer et al., 2024; Hendriks et al., 2024; Jeon et al., 2025; Huang et al., 2025). Current treatments such as surgery, radiotherapy, and chemotherapy have shown clinical benefits (Bahmani et al., 2024; Elghali et al., 2024; Deng et al., 2025; Cheng et al., 2026). However, drug resistance and severe side effects remain major challenges (Gong et al., 2024; Joukhan et al., 2024; Wang Y. H. et al., 2025; Wong et al., 2025). This is especially true for the A549 adenocarcinoma subtype, which is highly aggressive, prone to metastasis, and often develops resistance to conventional therapies (Tièche et al., 2019; Melocchi et al., 2021; Xin et al., 2025; Xu L. et al., 2025). These features make it a key focus, and a significant hurdle, in clinical oncology (Xu Y. et al., 2025; Zhao et al., 2025; Dwiranti et al., 2026; Zhao et al., 2026). PDT offers a promising alternative (Liu and Qian, 2024; Urazaliyeva et al., 2024; Zheng et al., 2024a; Monisha et al., 2025; Haghighi et al., 2026). It is minimally invasive, has low systemic toxicity, can be repeated, and does not induce drug resistance (Bora et al., 2024; Mishra et al., 2024; Porubský et al., 2024). In PDT, three components are required: a light source, a photosensitizer, and oxygen (Liu et al., 2025; Yeo et al., 2024). Light excites the photosensitizer, which then transfers energy to oxygen, generating reactive oxygen species (ROS) that kill tumor cells (Zaman et al., 2025; Negi and Venkatesh, 2026; Xiong et al., 2026).

Porphyrins and their derivatives are the most widely used photosensitizers in PDT (Chen S. et al., 2024; Glowacka-Sobotta et al., 2024; Huang et al., 2026). They exhibit excellent photophysical properties but suffer from poor water solubility, aggregation-induced fluorescence quenching, and an inherent trade-off between fluorescence quantum yield and singlet oxygen generation (Olubowale et al., 2024; Patra et al., 2025; Amirinejad and Shiri, 2026; Dai et al., 2026). These limitations hinder their use in combined diagnosis and therapy. To address these issues, researchers have taken two main approaches. First, the researchers redesigned photosensitizer molecules by building donor–acceptor systems or using supramolecular assembly to enhance performance (Zhao et al., 2023; Zhu et al., 2019). Second, they developed smart delivery platforms that respond to multiple features of the A549 tumor microenvironment, such as overexpressed MMP-2, acidic pH, hypoxia, and high ROS levels, to achieve precise targeting and controlled activation (Yang et al., 2022; Xu et al., 2024; Shu et al., 2021; Yang et al., 2023). Despite these advances, standalone PDT still faces intrinsic barriers, including tumor hypoxia (He and Ma, 2024; Nene and Abrahamse, 2024; Zhang P. et al., 2024; Li et al., 2026), antioxidant defense systems (Pang et al., 2024; Wang S. et al., 2024; Chen L. et al., 2025), and reliance on a single cell-killing mechanism. To overcome this, researchers have integrated PDT with other therapeutic modalities, such as chemotherapy (Guo et al., 2024; Li Y. et al., 2024; Cao et al., 2025; Zhou et al., 2025; Zou et al., 2026a), starvation therapy (Meng et al., 2024; Wu X. et al., 2024; Zheng et al., 2024b; Zhu et al., 2025), ferroptosis (Dong et al., 2024; Li J. et al., 2024; Zhang L. et al., 2024), and pyroptosis (Chen T. et al., 2024; Gao et al., 2024; Xu M.-Y. et al., 2025; Yu et al., 2025). This review demonstrates that the combination strategies create synergistic effects through cascade amplification, metabolic pathway coupling, and interconversion of cell death modes, demonstrating significantly enhanced efficacy in A549 models (Jiao et al., 2025; Xiao et al., 2022). Numerous studies confirm that such combination strategies hold great potential to break through the limitations of conventional PDT (Kim et al., 2024; Zhao D. et al., 2024; Yang et al., 2025).

Building on these synergistic approaches, innovative delivery vehicles are accelerating the clinical translation of photosensitizers. For example, facultative probiotic-based live carriers act as “biological navigators” to deliver chlorin e6 (Ce6) deep into the hypoxic core of A549 tumors, greatly improving drug penetration and accumulation (Guo et al., 2025). Biomimetic cell membrane vesicles anchor photosensitizers directly onto tumor cell membranes via membrane fusion, avoiding the immunogenicity of synthetic materials (Gao et al., 2024; Wang Y. et al., 2024). All-API (active pharmaceutical ingredient) nanoparticles, containing 100% therapeutic payload, eliminate the low drug-loading and excipient toxicity problems of traditional nanomedicines, showing excellent biocompatibility and spatiotemporally controlled therapeutic outcomes in A549 tumor models (Fang et al., 2023).

**FIGURE 1:**
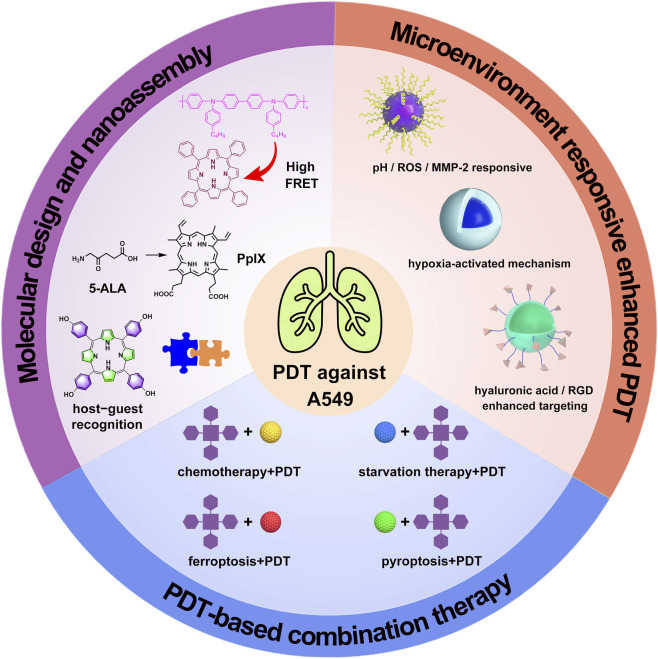
Schematic illustration of porphyrin-based photosensitizers for A549 tumor therapy, highlighting enhanced efficacy through molecular optimization, microenvironment-responsive design, and multimodal combination therapy.

In summary, porphyrin-based photosensitizers for A549 lung cancer PDT have rapidly evolved, from molecular optimization and microenvironment-responsive design to multimodal combination therapy and advanced delivery systems (Figure 1). Based on these challenges and opportunities, this review follows a progressive logic from fundamental mechanisms to clinical applications, systematically examining the developmental trajectory of porphyrin-based photosensitizers in A549 lung cancer PDT: first elucidating the basic mechanisms of PDT and molecular design strategies of porphyrin photosensitizers, then analyzing the construction principles of tumor microenvironment-responsive delivery systems, followed by exploring innovative strategies of multimodal synergistic therapy, and finally summarizing the challenges and future directions for clinical translation. This review systematically summarizes recent progress across these areas, with a focus on how molecular engineering, nanotechnology, and bio-inspired carriers can overcome current bottlenecks. We aim to provide a clear reference for future research and clinical translation in this field.

## 2 Phototherapeutic mechanism of porphyrin-based photosensitizers

**FIGURE 2:**
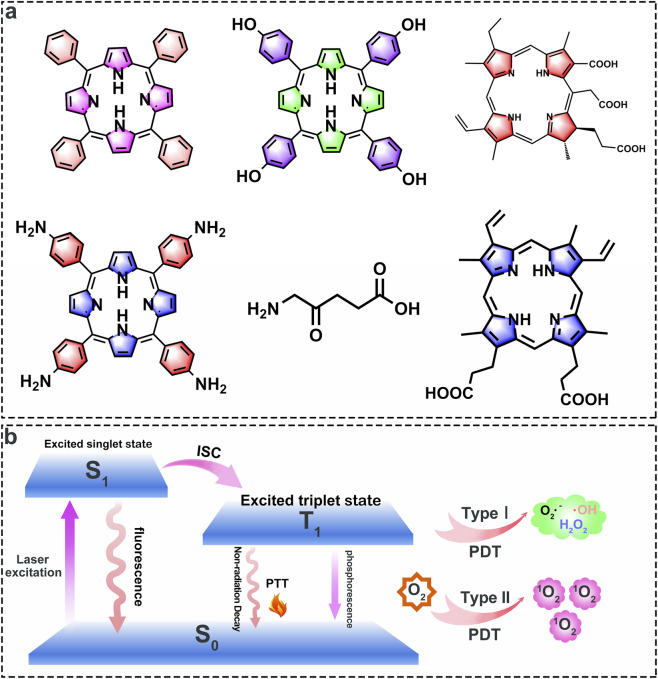
**(a)** Molecular structures of five porphyrin-based photosensitizers and 5-ALA. **(b)** Schematic illustration of the phototherapeutic mechanism of porphyrin-based photosensitizers.

Porphyrins are a class of conjugated organic molecules formed by linking pyrrole rings through methine bridges (Tanaka and Osuka, 2015; Park et al., 2021). They exhibit structural diversity and good biocompatibility (Zou et al., 2017; Tian et al., 2020; Zou et al., 2026b). The literature reviewed here includes various porphyrin derivatives, as well as precursors like 5-aminolevulinic acid (5-ALA), which can be metabolized *in vivo* to generate porphyrins (Figure 2a). This structural variety enables the development of diverse porphyrin-containing nanoplatforms for biomedical applications in both antimicrobial and anticancer therapies (Galstyan et al., 2020; Yamada and Komatsu, 2023). These five porphyrin molecules can be obtained through laboratory chemical synthesis, while 5-aminolevulinic acid (5-ALA), as a naturally occurring biomolecule, can be prepared either through chemical total synthesis or microbial fermentation. Laboratory synthesis ensures high purity of the porphyrin molecules. Furthermore, these five porphyrin molecules possess distinct functional group structures that enable tight integration with corresponding delivery systems and therapeutic strategies, thereby achieving optimal system design and potentially enhanced PDT efficacy. Currently, these five porphyrin molecules remain at the preclinical research stage. In contrast, 5-ALA has already received formal approval in multiple countries worldwide as a clinically applied pharmaceutical molecule and can be directly utilized for photodynamic therapy in clinical practice. 5-ALA sequentially condenses and cyclizes along the heme synthesis pathway to generate protoporphyrin IX (PpIX). In normal cells, PpIX rapidly combines with iron to form heme. Due to enzymatic activity defects in tumor cells, PpIX cannot be properly converted and accumulates, subsequently producing reactive oxygen species upon light irradiation to achieve photodynamic killing.

The therapeutic effect of porphyrins relies on their ability to generate highly oxidative ROS that suppress cancer cell survival (Zou et al., 2024; Chen M. et al., 2025; Zou et al., 2025). In PDT, the process begins when light excites the photosensitizer to its singlet state (Zheng et al., 2026). Through intersystem crossing, this singlet-state photosensitizer converts to a longer-lived triplet state. From this triplet state, two main reaction pathways can occur. In Type I PDT, the triplet photosensitizer transfers electrons to surrounding biomolecules or oxygen, producing radicals such as superoxide anion (O_2_•^-^) and hydroxyl radical (•OH). In Type II PDT, the triplet photosensitizer transfers energy directly to molecular oxygen, generating singlet oxygen (^1^O_2_) (Figure 2b). These reactive oxygen species then interact with key cellular components, including proteins, polysaccharides, and lipids, causing oxidative damage and functional loss. This ultimately triggers cancer cell death via apoptosis or necrosis, thereby inhibiting tumor growth. In the hypoxic microenvironment characteristic of A549 tumors, Type I reactions often predominate, which holds significant pharmacological importance for overcoming the therapeutic limitations of conventional Type II reactions (requiring O_2_ to produce ^1^O_2_) under hypoxic conditions. This mechanistic shift not only enhances the cytotoxic efficacy of photodynamic therapy in the hypoxic core regions of solid tumors but also provides crucial pharmacological guidance for designing next-generation porphyrin-based photosensitizers. Moreover, Triplet-state photosensitizers can also return to the ground state via non-radiative pathways, such as vibrational relaxation, releasing heat in the process, which constitutes a potential photothermal mechanism for certain porphyrin-based photoactive materials.

Porphyrin derivatives exhibit multiple absorption bands in the 400–700 nm range: the Soret band (420–440 nm) represents a strong absorption peak, and excitation within this region yields the highest ^1^O_2_ quantum efficiency. In contrast, the Q bands (500–750 nm) consist of several weaker absorption peaks; under identical irradiation conditions, excitation in this range results in significantly lower ^1^O_2_ generation due to reduced molar absorptivity. To highlight how varying illumination conditions can significantly influence phototherapeutic outcomes, we have systematically compiled key information from the representative studies reviewed herein into Table 1. This includes the material names, carrier design strategies, light source parameters (e.g., excitation wavelength, power density, and irradiation duration), and the distinct therapeutic advantages demonstrated by each nanoplatform in photodynamic therapy. This table not only facilitates a direct comparison of photoresponsive characteristics across different systems but also underscores the critical importance of synergistically optimizing both light delivery and carrier design to enhance therapeutic efficacy.

**TABLE 1:** Synthesis of various porphyrin-based or porphyrin precursor (5-ALA) nanoplatforms for enhanced therapy of A549 lung cancer.

Nanosystem	Treatment/Excitation light source		Therapeutic property	Ref
	Treatment	Excitation light source		
ADS254BE/H_2_TPP	PDT	blue LED light (374 nm), 0.06 W cm^-2^	Size≈26 nm, spherical morphology, D–A structure, FRET mechanism, image-guided PDT, conjugated polymer as the carrier	Zhao et al. (2023)
TPP@NPs	PDT	660 nm, 300 mW cm^-2^	Spherical NPs, ERGDS-containing peptide for targeting, loading efficiency = 65%, supramolecular peptide as the carrier	Zhu et al. (2019)
DSPM@Ce6@Gd	PDT	633 nm helium−neon (He−Ne) laser (50 mW/cm^2^, 2 min)	Ce6-loading efficacy = 6.1%, Size≈157.1 nm, spherical morphology, EPR/MMP2 targeting, NIRF/MR imaging DSPE-PEG2000 as the carrier	Yang et al. (2022)
PEG-M-PPMT	PDT/chemotherapy	635 nm, 500 mW/cm^2^, 10 min	Nanosphere, Size≈135.1 nm, loading efficacy (2.7%–2.8% SRF and 1.7%–1.9% Ce6, pH/ROS/MMP-2 triple-responsive, block copolymer as the carrier	Shu et al. (2021)
HANGs Ce6	PDT	660 nm, 0.5 W/cm^2^, 20 min	Size≈96 nm, zeta = −16 mV, hyaluronic acid-targeting, hypoxia-activated and enhanced FL, copolymer as the carrier	Yang et al. (2023)
SPCCM Ce6/DOX	PDT/immunotherapy	660 nm, 0.2 W/cm^2^, 2 min	Size≈122 nm, zeta = −19.47 mV, pH-Responsive Release, tumor cell membrane encapsulation	Xu et al. (2024)
PpIX-1-DG NPs	PDT/chemotherapy	650 nm, 1 W/cm^2^, 2 min	Size≈80.9 ± 11.2 nm, zeta = −2.6 ± 0.2 mV, GSH-responsive, amphiphilic molecules as the carrier	Jiao et al. (2025)
RCA-Chol@CHG	PDT/starvation therapy	650 nm He-Ne laser, 50 mW/cm^2^	Size≈615 nm, zeta = −23.9 mV, nanoflower, glucose decomposition and O_2_ production, hydrogel as the carrier	Liu et al. (2023)
CH/DF	PDT/ferroptosis	660 nm, 0.75 W/cm^2^, 1 min	Size≈350 nm, O_2_ generation for hypoxia relief, GSH depletion, aptamer-mediated targeting delivery, DNA nanozyme as the carrier	Xiao et al. (2022)
AFeC FANDs	PDT/CO therapy/ferroptosis	635 nm, 0.3 W/cm^2^, 10 min	bowl-like structures, diameter≈70 nm, zeta = −16.1 mV, pH-responsive	Fang et al. (2023)
EcN-WCS@Ce6 (EWC)	PDT	660 nm, 0.1 W/cm^2^, 5 min	rod-like shape, zeta = −16.1 mV, EcN as a carrier for targeting delivery	Guo et al. (2025)

Abbreviations: EcN (*Escherichia coli* Nissle 1917); SRF (sorafenib).

## 3 Molecular design and nanoassembly for enhanced PDT

**FIGURE 3:**
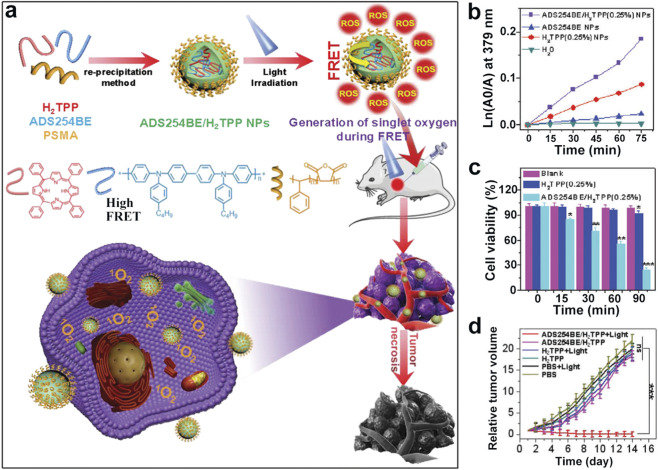
**(a)** Schematic illustration of ADS254BE/H_2_TPP NP preparation and ROS generation via FRET for A549 tumor suppression. **(b)** Singlet oxygen detection results of ADS254BE/H_2_TPP NPs compared with various controls. **(c)** Cytotoxicity assessed by MTT assay. **(d)** Tumor growth inhibition in different treatment groups. Reproduced with permission from (Zhao et al., 2023). Copyright (2023), Wiley-VCH GmbH.

The hydrophobic structure of porphyrin molecules leads to poor water solubility and a strong tendency to aggregate, which greatly limits their phototherapeutic efficacy. In addition, porphyrins are typical organic small molecules that suffer from aggregation-caused quenching. This results in an inherent conflict: high fluorescence intensity often comes at the expense of singlet oxygen generation, and *vice versa*. Resolving this trade-off is essential for enabling fluorescence imaging–guided PDT. To tackle these issues, the Tang group developed a donor–acceptor (D-A) system by encapsulating the porphyrin H_2_TPP, as the acceptor, into the conjugated polymer ADS254BE, forming stable nanoparticles (Figure 3a) (Zhao et al., 2023). Their studies showed that, through a fluorescence resonance energy transfer (FRET) mechanism, this system boosted the fluorescence intensity of H_2_TPP by approximately 11.4-fold and increased its singlet oxygen yield by nearly twofold (Figure 3b). When compared with free H_2_TPP, the D-A nanocomposite demonstrated significantly stronger inhibition of A549 cancer cell viability (Figure 3c). In both cultured A549 cells and xenograft tumor models, the nanoparticles exhibited bright red emission, excellent singlet oxygen production, and robust photodynamic therapeutic effects (Figure 3D). In this work, the conjugated polymer ADS254BE serves as the donor, absorbing excitation light energy and efficiently transferring it to the acceptor H_2_TPP molecules via FRET mechanism. This not only significantly enhances the fluorescent emission of H_2_TPP but also maintains and optimizes its intersystem crossing process, thereby preserving or even enhancing the singlet oxygen generation efficiency. This mechanism cleverly circumvents the inherent trade-off in conventional photosensitizers where fluorescence enhancement inevitably leads to decreased singlet oxygen production, providing a new strategy for achieving both efficient imaging and potent therapeutic effects simultaneously. More importantly, although the *in vivo* environment presents potential limiting factors such as limited light penetration depth and oxygen availability, this study circumvented systemic delivery barriers through intratumoral administration and significantly enhanced photosensitizing efficiency via the D–A architecture, thereby achieving effective translation of *in vitro* performance into *in vivo* therapeutic efficacy. This work presents a practical molecular engineering strategy to simultaneously improve solubility, reduce aggregation, and balance fluorescence with reactive oxygen generation, offering a promising approach for effective PDT against A549 lung adenocarcinoma.

**FIGURE 4:**
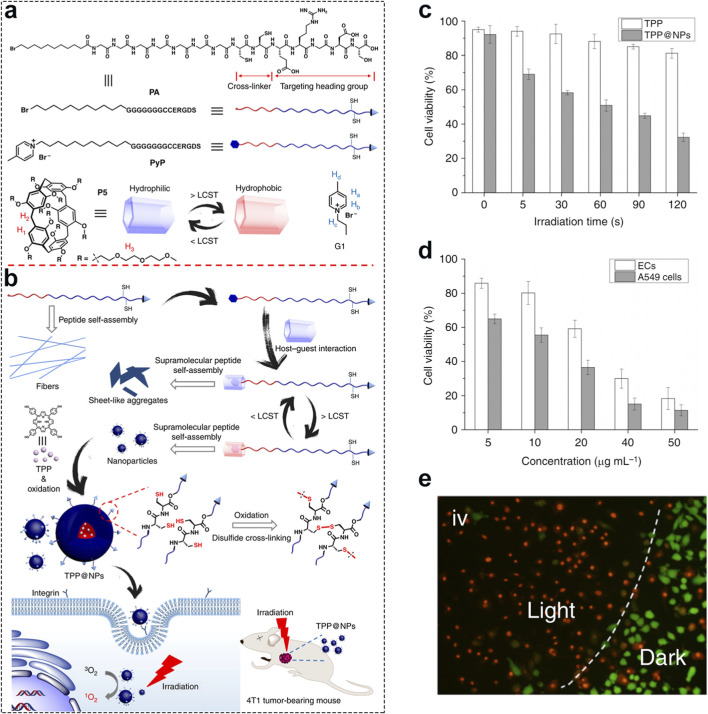
**(a)** Molecular structure of the supramolecular peptide. **(b)** Schematic illustration of supramolecular peptide self-assembly and porphyrin loading for antitumor activity. **(c)** Comparison of A549 cell viability after treatment with TPP versus TPP@NPs. **(d)** Cytotoxicity of TPP@NPs in normal cells versus A549 cancer cells. **(e)** Live/dead staining of A549 cells treated with TPP@NPs under light irradiation. Reproduced with permission from (Zhu et al., 2019). Copyright (2019), Springer Nature.

Although covalent assembly strategies can effectively enhance photosensitizer performance, the introduction of conjugated polymers often adds complexity to the system. Some researchers have found that peptides can directly bind to photosensitizers and drive self-assembly, offering a simpler alternative. However, conventional peptide-based photosensitizers usually require multi-step chemical modifications and precise control over assembly, which limits their practical use. To address this, the Huang group adopted a supramolecular approach (Zhu et al., 2019). They constructed a supramolecular peptide using host–guest recognition based on pillar (Gao et al., 2026) arene (Figure 4a) (Zhu et al., 2019). This design eliminated the need for complex covalent synthesis and purification. Instead, the components underwent programmed self-assembly to form stable nanoparticles (Figure 4b). These nanoparticles featured an ERGDS targeting sequence on their surface and a hydrophobic core, enabling efficient encapsulation of the photosensitizer tetra (hydroxyphenyl)porphyrin (TPP), yielding the TPP@NPs nanoplatform (Figure 4b). The authors then evaluated its cytotoxicity using the MTT assay, a widely accepted method for measuring a drug’s ability to inhibit cancer cell viability (Wang Z. et al., 2020). Results showed that TPP@NPs achieved significantly higher PDT efficacy in A549 cells than free TPP, thanks to enhanced cellular uptake mediated by the peptide component (Figure 4c). Moreover, the presence of the ERGDS motif conferred selective toxicity toward A549 cancer cells over normal endothelial cells (ECs) (Figure 4d). Live–dead cell staining provided further visual confirmation: under light irradiation, TPP@NPs effectively killed A549 cells (Figure 4e). This strategy simplifies formulation while preserving the biological activity of peptides, offering a new non-covalent route to optimize photosensitizer delivery.

## 4 Exploiting the A549 tumor microenvironment for precision PDT

**FIGURE 5:**
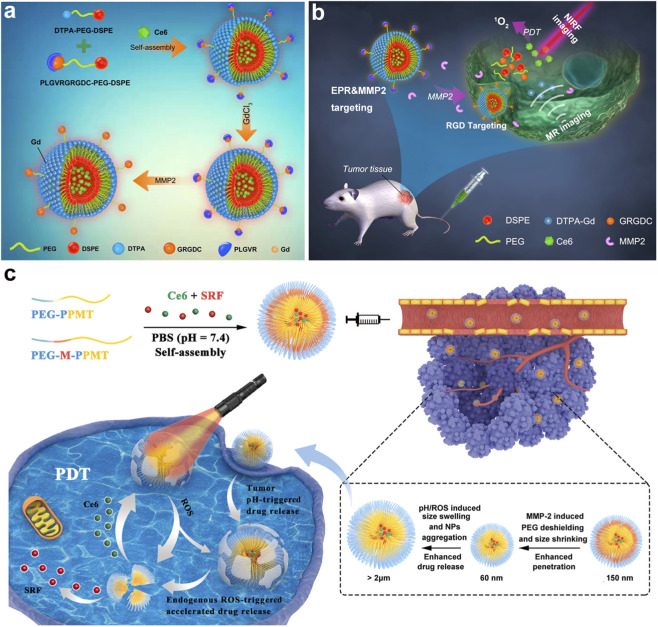
**(a)** Preparation steps of the nanoparticle platform. **(b)** Schematic illustration of the nanoparticle platform for PDT-mediated suppression of A549 cancer cell proliferation. Reproduced with permission from (Yang et al., 2022). Copyright (2022), American Chemical Society. **(c)** Preparation of the Ce6-based system and its tumor-responsive action under MMP-2, acidic pH, and ROS triggers. Reproduced with permission from (Shu et al., 2021). Copyright (2020), Elsevier Ltd.

Optimizing porphyrin photosensitizers at the molecular level is essential for improving their therapeutic efficacy. However, their distribution and activation efficiency *in vivo* are equally critical. The A549 tumor microenvironment exhibits several distinctive features, such as overexpression of matrix metalloproteinase-2 (MMP-2), acidic pH, hypoxia, and elevated ROS, which serve as ideal endogenous triggers for designing smart, responsive delivery systems (Xu H. et al., 2025). Combining molecular optimization with microenvironment-responsive strategies holds great promise for achieving “on-demand” activation and precise tumor targeting, thereby enhancing the selectivity of PDT. For example, the Zou group developed a self-assembled nanoparticle system that responds to MMP-2, which is highly expressed in A549 tumors (Figure 5a) (Yang et al., 2022). This system co-loaded gadolinium ions and the photosensitizer Ce6, and used an MMP-2-cleavable peptide (PLGVR) to mask a targeting ligand. Once the nanoparticles accumulated in the tumor, MMP-2 cleaved the peptide linker, exposing an RGD motif that promoted active uptake by A549 cells. This created a cascade response: first tumor localization, then cellular targeting (Figure 5b). Thanks to the dual loading of Gd^3+^ and Ce6, the system enabled effective NIRF/MR dual-modal imaging-guided PDT in A549 tumor–bearing mice (Figure 5b).

While single-enzyme targeting can improve specificity, the tumor microenvironment is complex and dynamic, often involving multiple overlapping signals. Relying on just one trigger may be insufficient. To address this limitation, the Liu group engineered PEG-M-PPMT nanoparticles that respond simultaneously to three stimuli: MMP-2, acidic pH, and ROS (Figure 5c) (Shu et al., 2021). MMP-2 cleavage removes the surface PEG layer, reducing particle size and enhancing tumor penetration. After cellular internalization, the acidic endosomal environment and high ROS levels further accelerate drug release (Figure 5c). Notably, laser irradiation during PDT generates additional ROS, which in turn promotes more nanoparticle disassembly, a positive feedback loop that significantly boosts therapeutic output. In A549 tumor–bearing mice, this triple-responsive system achieved a complete tumor eradication rate of over 70% (Figure 5c).

**FIGURE 6:**
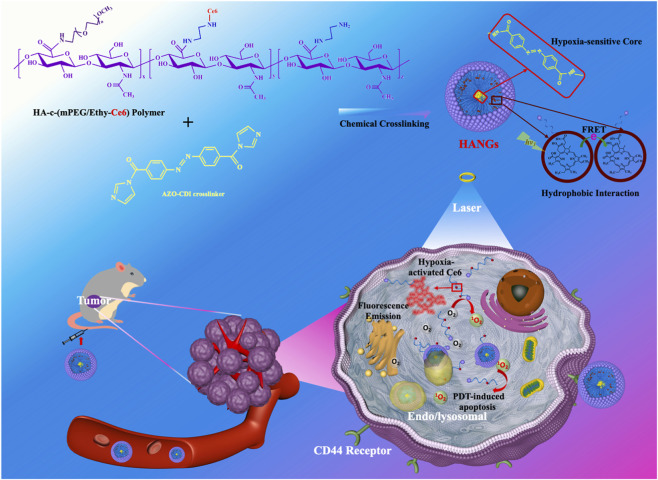
Preparation of the HANGs nanoplatform and its schematic illustration for antitumor action. Reproduced with permission from (Yang et al., 2023). Copyright (2023), Elsevier B.V.

Although multi-stimuli-responsive strategies have improved delivery precision, hypoxia in the core of A549 tumors remains a major barrier to effective PDT (Ma et al., 2026). A growing research focus is now on turning this “disadvantage” into an “activation switch.” For instance, Fu and colleagues developed a hypoxia-activatable hyaluronic acid-based nanogel (HANGs) that exploits tumor hypoxia as a trigger (Figure 6) (Yang et al., 2023). Under normal oxygen conditions, the fluorescence and ROS generation of Ce6 are effectively “off.” In the hypoxic core of A549 tumors, however, the azo crosslinkers in the nanogel are reduced, causing the gel to disassemble and “turning on” Ce6’s fluorescence and PDT activity (Figure 6). This system also leverages the specific binding between hyaluronic acid and the CD44 receptor, enabling efficient targeting of CD44-positive A549 cells. During treatment, it significantly suppressed tumor growth (Figure 6).

**FIGURE 7:**
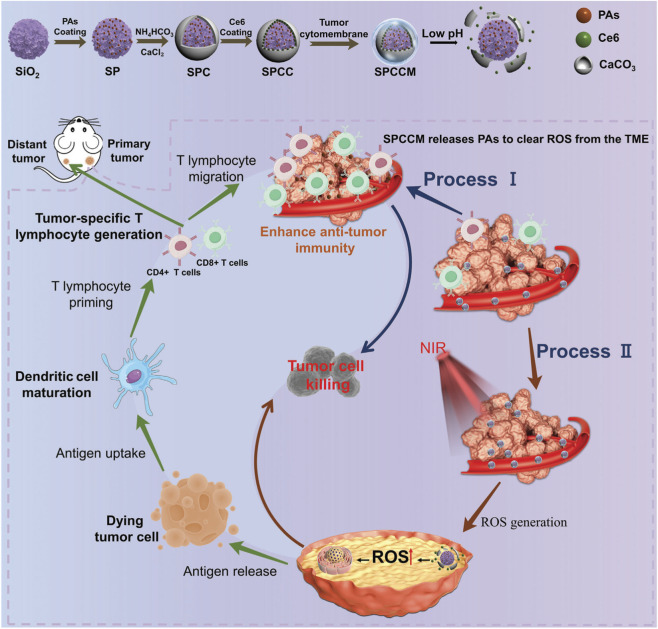
Preparation of the SPCCM nanoplatform and schematic illustration of its structural disassembly under mild acidic conditions to enable combined phototherapy and immunotherapy for antitumor effects. Reproduced with permission from (Xu et al., 2024). Copyright (2024), Wiley-VCH GmbH.

Achieving precise delivery and activation of photosensitizers in tumors can significantly improve PDT efficacy. However, the ROS generated during PDT also act as key signaling molecules. Strategically modulating ROS levels in the tumor microenvironment to enhance immune responses is an emerging area worth exploring. For example, the Gao group shifted focus from merely responding to the microenvironment to actively regulating its ROS balance. They developed a yolk–shell structured nanoplatform called SPCCM (Figure 7) (Xu et al., 2024). Its calcium carbonate shell degrades in the acidic tumor microenvironment (TME), releasing both the antioxidant proanthocyanidins (PAs) and the photosensitizer Ce6. PAs scavenge excess ROS to protect T lymphocytes, while light-activated Ce6 produces a high, localized burst of ROS that exceeds the threshold needed to trigger A549 cell apoptosis (Figure 7). This “clear first, then generate” sequential strategy successfully induced immunogenic cell death (ICD), leading to an 8-fold increase in dendritic cell activation (Figure 7). Given that immunotherapy has become a major focus in cancer treatment in recent years (Gao et al., 2020; Hu et al., 2020; Mao et al., 2023; Fan et al., 2025; Geng et al., 2025), this ROS-modulating approach, by linking PDT to immune activation, holds considerable translational potential. The study offers a new direction for highly effective phototherapy against A549 tumors.

While ROS modulation can boost immunity, most current strategies still rely on external laser irradiation. Limited tissue penetration of light remains a key bottleneck for treating deep-seated tumors (Tian et al., 2023; Zhou et al., 2024; Wang Z. et al., 2025). To overcome this, Xu and colleagues developed a self-illuminating nanoparticle based on bioluminescence resonance energy transfer (BRET) (Xu et al., 2019). The particle self-assembles from an amphiphilic polymer conjugate carrying luminol (the bioluminescent donor) and Ce6 (the fluorescent acceptor). In inflammatory or tumor microenvironments, endogenous ROS and myeloperoxidase (MPO) trigger the BRET process, enabling *in situ* generation of singlet oxygen without any external light. This system simultaneously supports deep-tissue imaging and PDT. In A549 tumor models, this self-illuminating platform demonstrated excellent tissue penetration, selective antitumor efficacy, and good biodegradability, effectively eliminating the dependence on external light sources that plagues conventional PDT. Although self-illuminating PDT enables precise treatment without external excitation, monotherapy still faces inherent limitations such as tumor hypoxia, antioxidant defenses, and reliance on a single cell-killing mechanism. Combining PDT with other therapeutic modalities offers a promising path forward to overcome these challenges.

## 5 Combining PDT with multimodal mechanisms to enhance A549 tumor killing

Numerous studies have shown that due to the high heterogeneity of malignant tumors, single-modality therapies often fail to deliver satisfactory outcomes (Zhang et al., 2016; Goswami et al., 2024; Wei et al., 2024; Zhang Q. et al., 2024). In recent years, research has increasingly shifted toward combination strategies to improve therapeutic efficacy (Wang N. et al., 2020; Yong et al., 2020; Tao et al., 2022; Qi et al., 2024; Gong et al., 2025a). Although smart, stimulus-responsive systems have greatly enhanced the tumor targeting and activation efficiency of porphyrin-based photosensitizers in A549 models, standalone PDT still faces inherent limitations, such as hypoxia, antioxidant defense mechanisms, and reliance on a single cell-killing pathway. Combining PDT with other treatment modalities, including chemotherapy, starvation therapy, and ferroptosis, not only overcomes these barriers but also enables synergistic “1 + 1>2” effects through multi-mechanistic crosstalk. This approach opens new avenues for potentially curative treatment of A549 tumors.

### 5.1 PDT + chemotherapy

**FIGURE 8:**
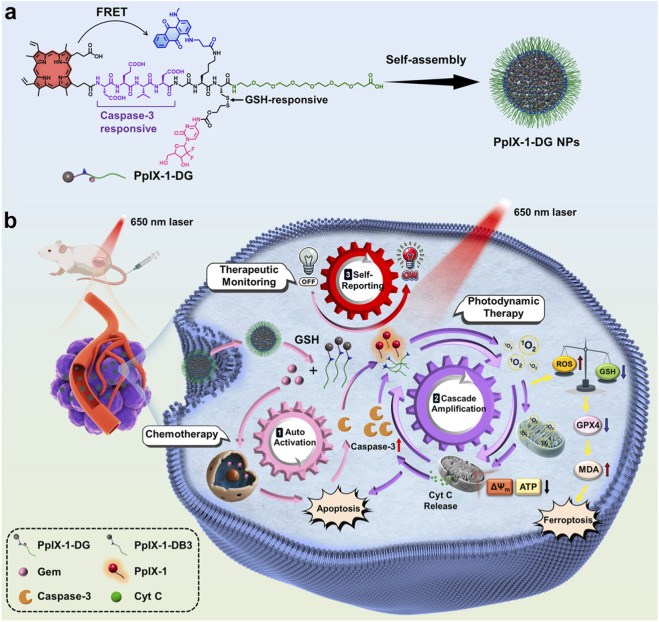
**(a)** Molecular structure of PpIX-1-DG and schematic illustration of its self-assembly into nanoparticles. **(b)** Schematic illustration of combined phototherapy, chemotherapy, and ferroptosis for antitumor effects upon 650 nm laser irradiation. Reproduced with permission from (Jiao et al., 2025). Copyright (2025), Elsevier Inc.

For instance, Zhong and colleagues designed a dual-responsive molecule, PpIX-1-DG, which responds to both glutathione (GSH) and caspase-3 (Figure 8a) (Jiao et al., 2025). This molecule self-assembles into a nanoplatform capable of deeply integrating chemotherapy and PDT (Figure 8a). In the high-GSH environment of A549 cells, the system first releases gemcitabine, initiating apoptosis and activating caspase-3. The activated caspase-3 then specifically cleaves the DEVD linker, restoring the activity of the photosensitizer protoporphyrin IX (PpIX) and triggering PDT (Figure 8b). The ROS generated by PDT further promotes apoptosis, which in turn elevates caspase-3 levels, creating a positive feedback loop: chemotherapy initiates the process, and PDT amplifies it. This system achieves three integrated functions: automatic activation, cascade amplification, and self-reporting. As a result, it demonstrates significantly enhanced antitumor efficacy in A549 cells (Figure 8b). In this system, the authors demonstrate that their engineered platform employs a multi-stage control mechanism, comprising GSH-responsive drug release, localized caspase-3 activation, and *in situ* restoration of the photosensitizer, to effectively prevent premature initiation of the cascade reaction, thereby ensuring therapeutic precision and safety.

### 5.2 PDT + starvation therapy

**FIGURE 9:**
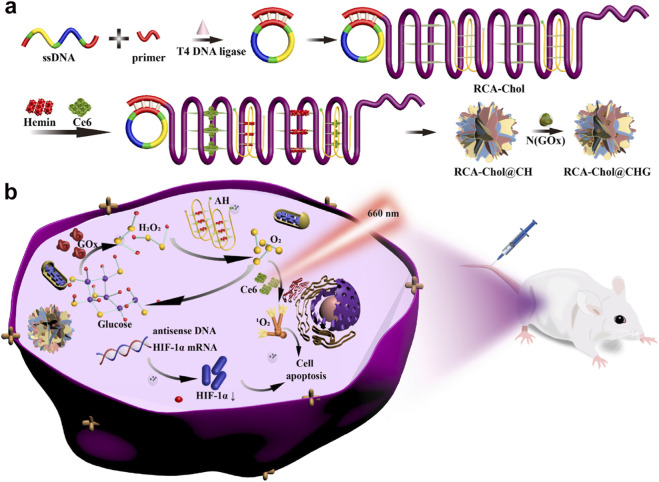
**(a)** Preparation steps of the RCA-Chol@CHG nanoplatform. **(b)** Schematic illustration of its antitumor action under 660 nm light irradiation. Reproduced with permission from (Liu et al., 2023). Copyright (2023), Elsevier Masson SAS.

Cascade reactions can effectively integrate chemotherapy with PDT. However, such strategies rely on the delivery of exogenous chemotherapeutic agents. A more “endogenous” approach would be to couple PDT directly with the tumor’s own metabolic pathways, potentially enabling self-sustained synergistic therapy. Starvation therapy is a strategy that suppresses tumor energy supply, such as by inhibiting glucose uptake or disrupting metabolic pathways, to induce cell death. For instance, certain nanoplatforms deplete glucose in the tumor microenvironment or inhibit ATP generation. The combination of photodynamic therapy with starvation therapy represents an emerging and highly promising anticancer strategy. For example, Li and colleagues developed a DNA hydrogel–based “domino” cascade reactor named RCA-Chol@CHG (Figure 9a) (Liu et al., 2023). This nanoplatform co-delivers glucose oxidase (GOx), heme, the photosensitizer Ce6, and an antisense sequence targeting HIF-1α, enabling combined starvation therapy and PDT. GOx consumes intratumoral glucose to produce H_2_O_2_. Heme then binds to G-quadruplex structures to form a catalase-like complex that converts H_2_O_2_ into O_2_, supplying the oxygen needed for efficient Ce6-mediated PDT. At the same time, downregulation of HIF-1α alleviates tumor hypoxia (Figure 9b). In A549 cells, this cascade system achieved strong synergy between starvation therapy and enhanced PDT, resulting in excellent antitumor efficacy and highlighting the potential of multi-target combination strategies.

### 5.3 PDT + ferroptosis

**FIGURE 10:**
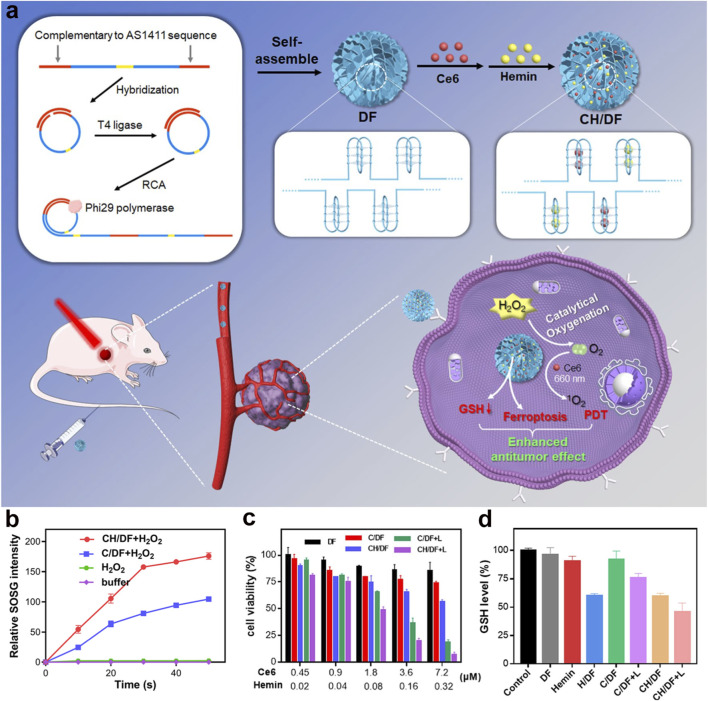
**(a)** Design strategy of the CH@DF nanoplatform and its schematic illustration for antitumor action. **(b)** Singlet oxygen generation capability of the CH@DF nanoplatform. **(c)** Cytotoxicity of the CH@DF nanoplatform. **(d)** GSH depletion ability of the CH@DF nanoplatform. Reproduced with permission from (Xiao et al., 2022). Copyright (2022), BioMed Central.

Ferroptosis is an iron-dependent form of regulated cell death driven by the lethal accumulation of lipid peroxides. In photodynamic therapy, photosensitizer-generated ROS can synergize with endogenous iron to amplify lipid peroxidation, thereby triggering ferroptosis. Integrating PDT with endogenous ferroptosis, a regulated form of iron-dependent cell death, could enable more sustained and synergistic antitumor effects. For instance, Zhou and colleagues developed a Ce6-loaded DNA nanozyme that combines PDT with ferroptosis to suppress A549 tumor growth (Figure 10a) (Xiao et al., 2022). The nanozyme incorporates the AS1411 G-quadruplex structure, which serves dual functions: it binds heme to form a catalase-like enzyme that converts endogenous H_2_O_2_ into O_2_, alleviating tumor hypoxia, and efficiently loads Ce6 for PDT (Figure 10a). More importantly, heme depletes intracellular GSH in A549 cells, while concurrently released Fe^2+^/Fe^3+^ ions trigger ferroptosis (Figure 10a). By simultaneously relieving hypoxia, depleting GSH, and activating two distinct cell death pathways, this nanozyme achieves strong synergy between PDT and ferroptosis, resulting in multimodal antitumor activity in A549 models. In their experiments, the authors used Singlet Oxygen Sensor Green (SOSG) to assess ROS generation. Results showed that in the presence of H_2_O_2_, the CH/DF nanozyme produced significantly more singlet oxygen, confirming its ability to catalyze H_2_O_2_ decomposition and generate O_2_ (Figure 10b). Cytotoxicity assays further demonstrated that CH/DF exhibited the strongest cell-killing effect under light irradiation compared with all control groups (Figure 10c). Additionally, GSH quantification confirmed that CH/DF effectively reduced intracellular GSH levels (Figure 10d). GSH depletion enhances PDT efficacy (Gong et al., 2025b; Jia et al., 2026; Liu J. et al., 2026; Liu S. et al., 2026; Wang et al., 2026; Yang et al., 2026), and ferroptosis represents a potent antitumor mechanism (Li et al., 2023; Wu J. et al., 2024; Zhang et al., 2026). This work proposes a synergistic strategy combining GSH depletion–enhanced PDT with ferroptosis induction, offering a novel approach for suppressing A549 lung cancer cells.

### 5.4 PDT + full-API nanodrug strategy

**FIGURE 11:**
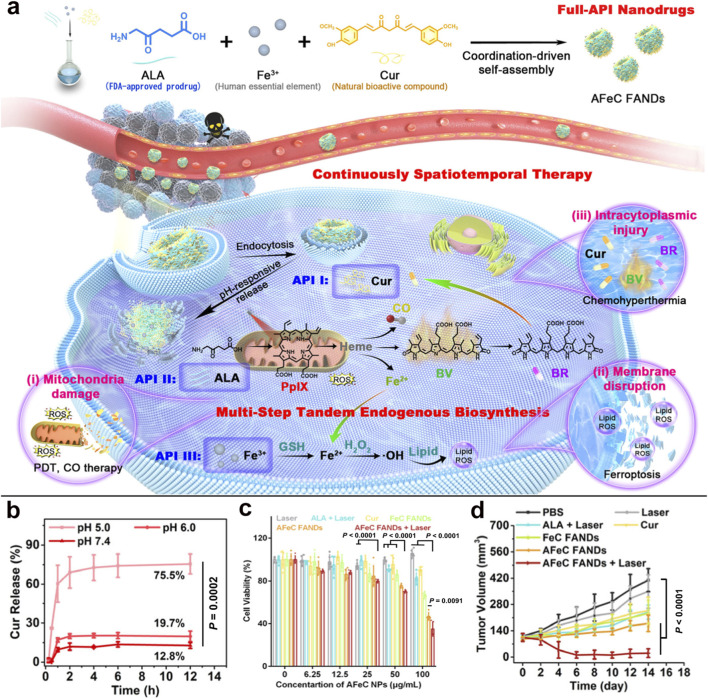
**(a)** Design strategy and schematic illustration of the AFeC FANDs nanoplatform for inhibiting A549 proliferation. **(b)** Curcumin release from AFeC FANDs under different acidic conditions. **(c)** Cytotoxicity of the AFeC FANDs nanoplatform. **(d)** *In vivo* tumor growth inhibition by AFeC FANDs compared with multiple control groups. Reproduced with permission from (Fang et al., 2023). Copyright (2023), Springer Nature.

Most combination strategies require integrating multiple functional components into a single nanocarrier. However, traditional nanocarriers often suffer from low drug-loading capacity and toxicity from inactive excipients, major hurdles to clinical translation. Developing safer and more efficient delivery systems has therefore become a critical challenge. To address this, Zhang and colleagues proposed a “full-API” nanodrug (FAND), representing an extreme simplification in delivery design (Fang et al., 2023). This system is entirely self-assembled from three FDA-approved or naturally occurring components: 5-aminolevulinic acid (ALA), Fe^3+^ (an essential human element), and curcumin, a natural product. No inert excipients are used, and the formulation achieves 100% active pharmaceutical ingredient (API) content (Figure 11a) (Fang et al., 2023). ALA is enzymatically converted into protoporphyrin IX (PpIX), a photosensitizing porphyrin derivative that can be activated by light (Bhattacharya et al., 2023; Chandratre et al., 2023; Ebrahimi et al., 2024). The FAND leverages endogenous biosynthetic pathways to link the photosensitizer PpIX, produced from ALA, with its downstream metabolites, including carbon monoxide (CO), Fe^2+^, and biliverdin. Upon a single laser irradiation, this cascade sequentially triggers mitochondrial damage, ferroptosis, and chemo-photothermal therapy, enabling multi-targeted and spatiotemporally coordinated treatment of A549 cells (Figure 11a). In detail, the researchers found that under mildly acidic conditions (pH = 5), the system released 75.5% of its curcumin payload (Figure 11b). Cytotoxicity assays confirmed that FAND strongly suppressed A549 cell viability under light irradiation (Figure 11c). *In vivo* tumor suppression studies further showed that the FAND + light group achieved the most potent antitumor effect among all tested groups, resulting in the smallest tumor volume (Figure 11d). Targeting specific organelles can significantly enhance phototherapy efficacy (Tao et al., 2025; Zhao X. et al., 2024). This system employs multiple mechanisms to induce mitochondrial damage and plasma membrane disruption, offering a novel strategy for photodynamic therapy against A549 lung cancer cells.

**FIGURE 12:**
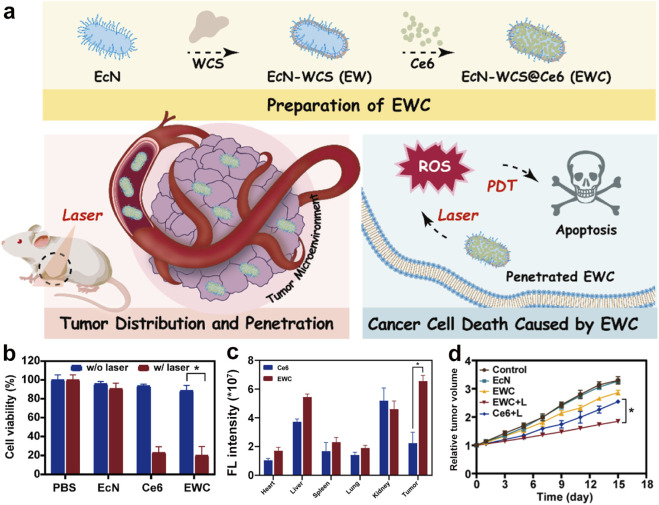
**(a)** Schematic illustration of EWC nanoplatform preparation and its enhanced cellular uptake for improved suppression of A549 proliferation. **(b)** Cytotoxicity of the EWC nanoplatform under light irradiation. **(c)** Fluorescence intensity distribution in various tissues after administration of EWC versus free Ce6. **(d)** Tumor volume changes following treatment with EWC or various control formulations. Reproduced with permission from (Guo et al., 2025). Copyright (2024), Elsevier B.V.

Beyond chemical self-assembly, using living biological entities as “active” carriers offers another innovative route to achieve safe and effective combination therapy (Lynch et al., 2022; Chen H. et al., 2025; Hu et al., 2025; Luo et al., 2025). For example, Miao and colleagues engineered the facultative probiotic *Escherichia coli* Nissle 1917 (EcN) as a live vector (Guo et al., 2025). They electrostatically attached the photosensitizer Ce6 to the bacterial surface using water-soluble chitosan, creating the EWC delivery system (Figure 12a) (Guo et al., 2025). This platform takes full advantage of EcN’s natural ability to selectively colonize hypoxic tumor regions, enabling precise delivery of Ce6 deep into the hypoxic core of A549 tumors. As a result, both penetration depth and drug accumulation within the tumor were significantly enhanced (Figure 12a). This “biological navigation” strategy effectively overcomes a major limitation of conventional nanoparticles, their poor penetration into deep tumor tissue. In A549 tumor–bearing mice, EWC demonstrated excellent antitumor efficacy. Cytotoxicity assays showed that the EWC platform strongly suppressed A549 cell viability under light irradiation (Figure 12b). Fluorescence imaging further confirmed that Ce6 accumulated more efficiently in tumors when delivered by live EcN, compared with free Ce6 or non-living carriers (Figure 12c). The combination of EcN-based vectors with PDT has emerged as one of the most promising strategies for designing advanced nanotheranostic platforms (Zhu et al., 2023; Zhang et al., 2025; Hsu et al., 2026; Yu et al., 2026). Thanks to this smart use of a living vector, the EWC system achieved superior tumor growth inhibition, resulting in significantly smaller tumor volumes than all control groups (Figure 12d). However, it should be noted that although EcN, as a generally recognized as safe probiotic, has demonstrated good tolerability in various diseases, the use of live bacterial carriers in cancer patients with severely impaired immune function should be approached with caution.

## 6 Conclusion

This review systematically summarizes recent advances in porphyrin-based photosensitizers for PDT of A549 lung cancer, covering four key dimensions: molecular optimization, smart microenvironment-responsive delivery, multimodal combination therapy, and innovative carrier design. At the molecular level, donor–acceptor architectures and supramolecular assembly strategies have effectively addressed the intrinsic trade-off between fluorescence quantum yield and singlet oxygen generation, as well as poor water solubility. In terms of microenvironment responsiveness, platforms that respond to multiple tumor-specific cues, such as MMP-2 overexpression, hypoxia, acidic pH, and elevated ROS, have enabled precise targeting and on-demand activation of photosensitizers. For combination therapy, strategies like chemotherapy–PDT cascade feedback loops, self-accelerating drug release, endogenous metabolic pathway coupling, and apoptosis-to-pyroptosis conversion have demonstrated strong synergistic effects. On the delivery front, living facultative probiotic carriers, biomimetic cell membrane vesicles, and 100% active pharmaceutical ingredient (API) nanodrugs have opened new pathways toward clinical translation. Despite these advances, significant challenges remain in translating porphyrin photosensitizers into clinical practice. The fundamental conflict between high fluorescence and high singlet oxygen yield has not been fully resolved. The D–A architecture provides a strategy to mitigate, rather than fully resolve, this inherent trade-off, and its performance remains constrained by factors such as molecular conformation, local microenvironment, and excitation parameters. Endogenous stimuli in tumors, such as MMP-2 or ROS, are often unevenly distributed and present at low levels, limiting reliable drug activation. Hypoxia further strengthens tumor resistance by upregulating HIF-1α and related pathways. Many current delivery systems suffer from complex manufacturing processes, poor reproducibility, and potential toxicity from metal-based components. Moreover, optimizing drug combinations and controlling treatment timing in multimodal regimens require deeper mechanistic understanding. Finally, a gap persists between preclinical studies and clinical application, largely due to the lack of standardized evaluation frameworks.

To address these issues, future research should focus on several key directions. First, at the molecular level, new donor materials should be explored in combination with porphyrin acceptors to develop next-generation derivatives that simultaneously achieve high fluorescence and high singlet oxygen yields. For example, constructing D–A-type porphyrin derivatives with strong donor–acceptor electronic effects or incorporating aggregation-induced emission (AIE) motifs to decouple the pathways of fluorescence and ROS generation. Non-covalent supramolecular approaches could also simplify peptide modification and improve the controllability of self-assembly, offering more efficient routes to performance enhancement. Second, smarter delivery systems are needed. These should integrate external triggers (e.g., light) with endogenous signals to drive drug release more reliably. Building on designs like hypoxia-activatable nanogels or triple-responsive (MMP-2/pH/ROS) platforms, future systems could incorporate cascaded responses to multiple microenvironmental cues, improving tumor penetration and delivery efficiency. Third, combination therapies must evolve from simple co-administration toward truly integrated mechanisms. Insights from chimeric peptide systems, featuring “automatic activation, cascade amplification, and self-reporting”, and all-API nanodrugs that harness endogenous metabolism can guide the rational design of synergistic interactions between different therapeutic modes. Fourth, carrier technologies need refinement. The preparation protocols for live probiotic vectors and cell membrane–based vesicles should be optimized to enhance drug loading stability and scalability. The all-API concept should be extended to more porphyrin systems to eliminate excipient-related toxicity at its source. Finally, clinical translation requires robust standardization. Comprehensive evaluation systems must be established to assess pharmacokinetics, biodistribution, and long-term safety in non-human primates. Equally important is exploring how porphyrin-based PDT can be integrated with existing clinical modalities, such as surgery, radiotherapy, and immunotherapy, to create practical combination regimens.

In summary, through coordinated advances in molecular design, delivery engineering, mechanistic synergy, and translational research, porphyrin-based photosensitizers hold strong promise for precision therapy of A549 lung cancer, and their path to the clinic is increasingly within reach.
